# The Protocol of Fixed Reconstruction for Severely Worn Teeth Combined with Anterior Deep Bite

**DOI:** 10.1155/2017/9378091

**Published:** 2017-08-23

**Authors:** Ya-Wen Zhao, Rong Gao, Hui-Qiang Sun

**Affiliations:** ^1^College of Stomatology, Shandong University, Jinan, Shandong 250012, China; ^2^Shandong Provincial Key Laboratory of Oral Biomedicine, Shandong 250012, China

## Abstract

Full mouth reconstruction is one of the most effective methods to restore severe worn teeth that have suffered reduced vertical dimension. Although the use of the overlay splint restoration for a trial period allowing the patient to adapt to an increased vertical dimension is the recognized method, the specific protocol from the transitional splint to the fixed reconstruction is yet to be established. This case report describes a 50-year-old female patient who has severely worn teeth combined with an anterior deep bite and chewing pain. The protocol of the treatment process is described.

## 1. Introduction

Severe tooth wear causes great distress to many patients who can be classified into 2 groups on the basis of decrease or no decrease in the vertical dimension. The decrease in the vertical dimension not only affects the function but also affects the appearance of teeth and the temporomandibular joint symptoms [1, 2]. For these patients, reconstruction is a beneficial treatment plan [3]. Of the many reconstruction treatment programs, the use of overlay splint restoration for a trial period that allows the patients to adapt to an increased vertical dimension is the recognized method [2, 4–9]. After the transition period of treatment, two types of permanent reconstruction are used: a removable permanent reconstruction and a fixed permanent reconstruction. It is very important during the transition process from the transitional splint to the permanent reconstruction that the permanent reconstruction should be the same as the vertical dimension and the anterior guidance of the anterior teeth as that in the period of transitional restoration [8, 9]. Although these are generally accepted treatments, the specific protocol from the transitional splint to the fixed reconstruction is yet to be established.

Introverted type deep overbite is usually caused during growth by genetics or by bad habits from the lingual inclination of the deep overbite of the anterior teeth. Because the position of the anterior teeth is incorrect, the posterior teeth do not have normal chewing function and protection function during the lateral and anterior protrusion movement of the anterior teeth. Consequently, the loss and wear of the posterior teeth will cause a deeper overbite, often accompanied by clinical symptoms of the temporomandibular joint [10, 11]. These factors make the treatment for severe worn teeth with anterior deep overbite even more complex.

Maxillofacial surgery, orthodontics, and restorative dentistry can be performed to help gain the vertical dimension and correct the anterior guidance in these patients [10]. Orthognathic treatment with an anterior segmental osteotomy has been recommended to obtain normal anterior guidance [12, 13]. Thus, interdisciplinary treatments, combining, for example, posterior restorations, implants with ceramic overlays of consumed, and abrased teeth and orthodontic treatment, should be considered. This paper reports the case of a patient with severe worn teeth and an anterior deep bite undergoing a fixed reconstruction. The protocol of the treatment process is described.

## 2. Case Presentation

**Figure 1:**
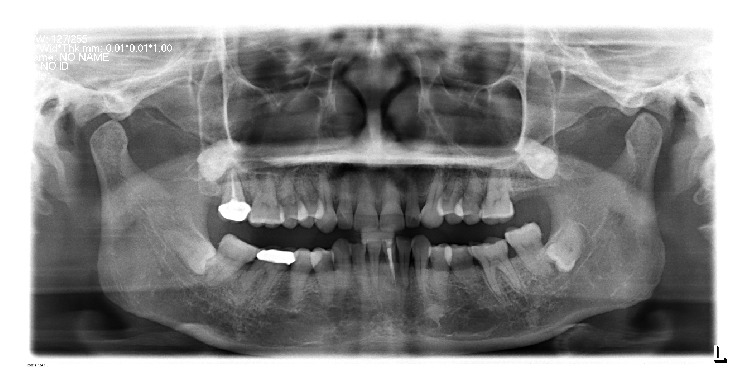
Digital panoramic tomography.

**Figure 2:**
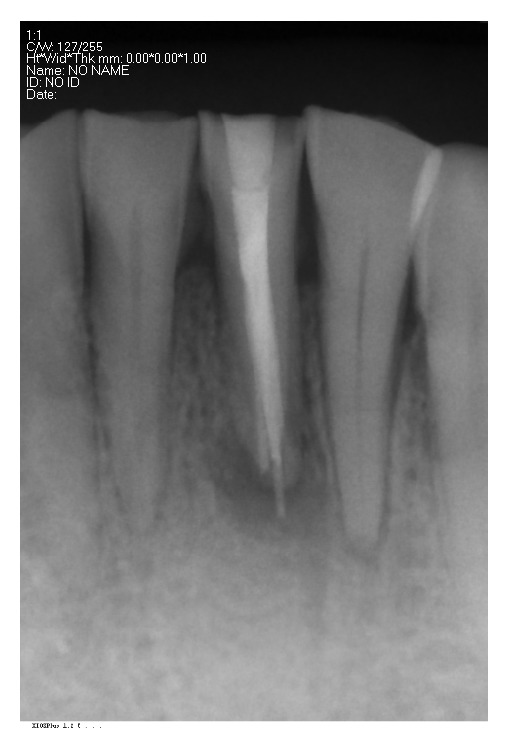
The digital periapical film of the left mandibular central incisor.

A 50-year-old female patient with chewing pain in the left posterior teeth visited the doctor. An intraoral examination revealed anterior teeth with a deep inward overbite and the loss of the normal protrusive guidance. The maxillary left second molar was empty, and the mandibular left second molar was extended by 5 mm. The maxillary right second molar and the mandibular right first molar were restored with crowns. The bilateral posterior teeth had severe attrition, exposed dentin, and probing sensitivity. The patient had a normal open-type, bilateral temporomandibular joint with mild click and no soreness of the mastication muscles. The lower vertical dimension of one-third of her face was 11 mm less than the vertical dimension of the middle one-third of her face, and the freeway space was 8-9 mm. The mandibular left first incisor had a labial apical fistula (Figures 1 and 2). An X-ray showed that the maxillary right second molar and the mandibular left first incisor had a root canal therapy, and there was an apical shadow in the mandibular left first incisor. The patient denied any history of bruxism.

The patient could not achieve the normal mandibular protrusion in the process of chewing food, with the anterior teeth inclined to a deep bite. The lateral movement was abnormal due to a collision between the mandibular left first incisor, whose position was the most anterior in the mandibular dentition, and the maxillary anterior teeth; thus, the patient could only exercise a linear mortar-and-pestle process when chewing food, which increased the posterior teeth load. In addition, the patient was missing a maxillary left second molar, causing more severe attrition of the left posterior teeth. Furthermore, the abnormal mortar-and-pestle mandibular movement also increased the burden of the temporomandibular joint. An interdisciplinary team including prosthodontist, oral and maxillofacial surgeon, orthodontist, and endodontist was formed to treat the patient. Alternative treatment projects were proposed to the patient. She refused orthodontic treatment due to the duration of treatment procedure. Meanwhile, surgery and restoration of the edentulous posterior region with an implant were offered. This option was also refused for the patient's fear of implant surgery. Therefore, prosthodontic treatment was chosen. The treatment plan was to regain the vertical dimension by reconstruction, then recover the anterior guidance through a mock-up of the anterior teeth, and finally remove the lock of the anterior teeth for full mouth rehabilitation.

In a conventional centric relation (CR) position when making a maxillary overlay splint, elevation is 5-6 mm at the posterior area, with adjustments to the occlusal contact points between the maxillary and mandibular dentition resulting in an anterior-posterior protected occlusion. The anterior teeth have no contact at the central occlusion, the bilateral posterior teeth have uniform contact. The posterior teeth have no contact at the anterior and lateral protrusive position and this establishes canine protected occlusion.

After 3 months, the patient had adapted to the new vertical dimension of occlusion (VDO) and anterior guidance. The occlusal relationship was stable without joint and muscle symptoms, and definitive restoration was started. During the 3-month trial period, the endodontic practitioner treated the mandibular left first incisor with root canal therapy and performed periapical surgery, which revealed a 5 mm apical longitudinal crack. The endodontist resected the apical granulation tissue and cut the 5 mm apical root.

**Figure 3:**
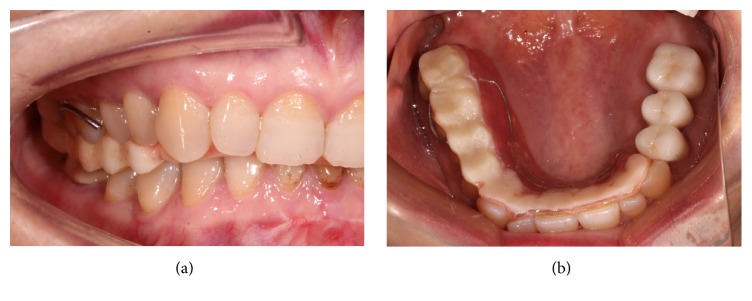
The right 2/3 removable overlay splint on the maxillary teeth.

**Figure 4:**
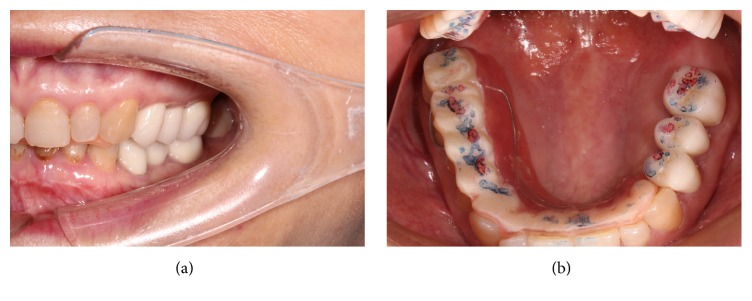
The completed ceramic crowns of the left posterior teeth. The red marks indicate the centric occlusion; the blue marks show the protrusive occlusion.

The prosthesis includes 3 steps. The first step involves the left maxillary and mandibular posterior fixed prosthesis. Cut the overlay splint into 2 sections from the distal border of the maxillary left canine. Continue to wear the right two-thirds part of the splint (Figures 3(a) and 3(b)) to support the transition relation, transfer the vertical dimension and the occlusal relation record as determined by the adapted trial treatment to the articulator, make the fixed reconstruction of the maxillary and mandibular posterior teeth with full porcelain crowns (Figure 4(a)), and operate conventional occlusal adjustment (Figure 4(b)).

**Figure 5:**
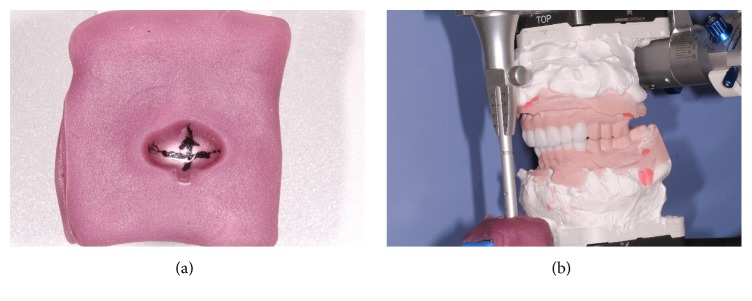
The fabrication of the personalized anterior guidance disk and the diagnostic wax-up of the anterior teeth.

**Figure 6:**
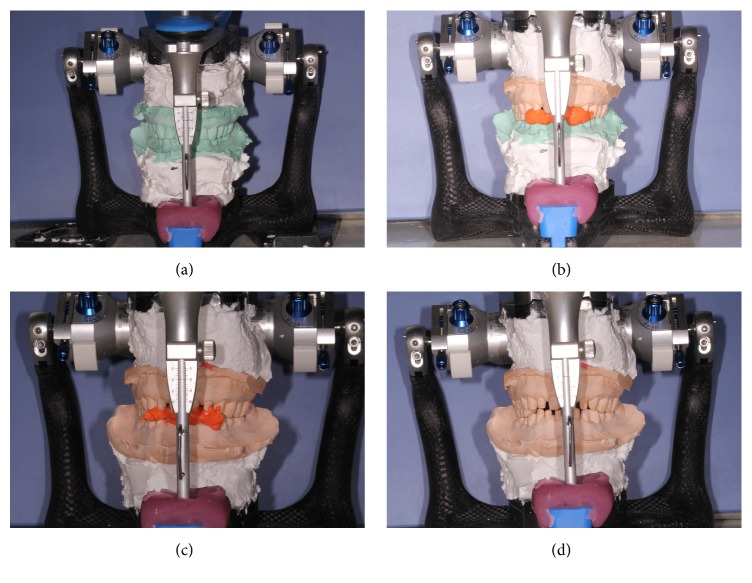
Mounting the articulator with the maxillomandibular relations using relation recording silicone rubber alternately.

**Figure 7:**
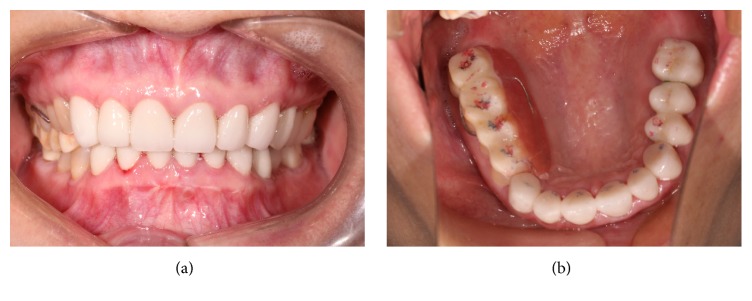
The anterior teeth were treated using a fixed restoration according to the anterior guidance of the transitional occlusal splint. The red marks indicate the centric occlusion; the blue marks show the protrusive occlusion.

The second step involves the anterior fixed prosthesis. Make an impression of the maxillary and mandibular dentition with the maxillary remaining two-thirds part of the splint worn in the mouth, mount on an articulator using a face bow, transfer the protrusive condylar guide inclination and the lateral condylar guide inclination using the anterior and lateral protrusive jaw position recording silicone rubber, and make a personalized anterior guidance disk with lightly cured resin (Figure 5(a)). According to this anterior guidance, make the maxillary and mandibular anterior teeth wax-up (Figure 5(b)) and then remove the anterior part of the rest of the splint. According to the tooth preparation silicone guide to prepare the maxillary and mandibular incisors, make a polyether impression, make the temporary crowns, mount the maxillary and mandibular models in turn on the articulator (Figures 6(a)–6(d)), and eventually place the full ceramic crowns (Figure 7(a)). The maxillary and mandibular anterior fixed reconstruction retains the anterior guidance of the transitional splint (Figure 7(b)).

**Figure 8:**
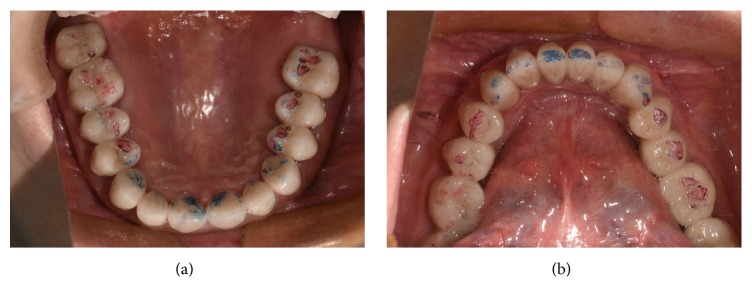
The fixed restoration of the right maxillomandibular posterior teeth according to the anterior guidance of the transitional overlay splint. The red marks indicate the centric occlusion; the blue marks show the protrusive occlusion.

The third step is the removal of the left segment of the splint. This step completes the ceramic crown restoration for the right maxillary and mandibular posterior teeth in accordance with the occlusal relationship (Figure 8).

**Figure 9:**
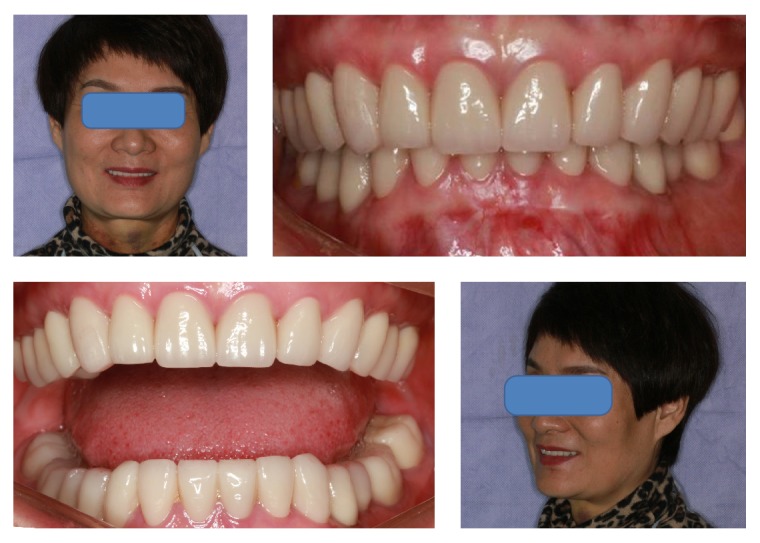
Frontal and lateral views of the finished dental restoration.

After the transitional restoration of the normal vertical dimension and the anterior guidance adaptation by the splint and the eventual full mouth rehabilitation using the ceramic crowns, the patient's left masticatory pain disappeared. After 4-month follow-up, examination was done and the minor occlusion registration was checked; there was no other discomfort and the denture appearance and shape were improved (Figure 9). It was recommended that the patient have an implant to restore the left maxillary second molar, and she planned to have the treatment later.

## 3. Discussion

The patient's needs, finances, motivation, and time dictate prosthodontic treatment options for patient with generalized tooth wear [14]. When making a treatment plan, the age of the patient should be considered. In clinical choices, life expectancy, duration, patient comfort, compliance, and esthetics are considered. The anterior deep bite can be treated using orthodontic surgery, orthognathic surgery, prosthetic treatment, or a combination of the 3 [11, 15]. The first 2 methods are, however, time-consuming, and there are limitations of indication [16]. Direct composite restorations are relatively cheap, esthetic, and easy to operate. Well, it is hard to test the wear resistance after restoration to find a fixed reference point in mouth or on study cast [17]. In this case, the patient was relatively old with severely worn posterior teeth and the vertical dimension was significantly reduced. The patient had few temporomandibular symptoms and preferred short duration of treatment and superior long-term follow-up result; hence, the prosthetic treatment was more suitable.

With regard to the protocol for occlusal reconstruction, Brown [7] had a classic discussion that describes the definite restoration process for patients who have a splint in the maxillary dentition: make 2 maxillary canine preparations and temporary crowns first, perform a complete restoration and establish the primary anterior guiding and canine protection, and finally perform the maxillary anterior incisor prosthesis and the restoration of the appearance of the anterior teeth. The restoration of the last 2 posterior teeth is then performed to allow the two maxillary second molar teeth and the canines establish a stable occlusal relationship, in the order of the first molar, the second premolar, and the first premolar to make the crowns. For patients whose splint transition restoration is in the mandibular dentition, when making the definite restoration, do not restore the anterior teeth, directly according to the mandibular splint to restore both of the mandibular second molar teeth first and then to restore both first molars and then restore the premolars; this is his protocol program. Brown believes that if one side restoration is made first, the occlusal relation transferring from the transition splint to the definite denture is not accurate, but our method is to begin to restore the posterior teeth from one side. In comparison, Brown's method has the following shortcomings: first, he did not separate the space equally. For the maxillary splint, he made only the maxillary fixed restoration and the single mandibular fixation for the mandibular splint transition. Second, he did not restore the worn teeth of the opposite dentition and did not play a protective role. Each of our methods involves restoring the maxillary and mandibular jaw at the same time, and the end of the fixed prosthesis can be a good bisection of the increased occlusal gap. Third, in Brown's method, the order is the canine, incisor, second molar, first molar, and premolar. This increases patient visit times, and the return period is longer. In our order, the first step is on one side of the maxillary and mandibular posterior teeth, and the second step involves the maxillary and mandibular anterior teeth. The third step is on the other side of the maxillary and mandibular teeth, to finish the prosthesis.

One side of the posterior teeth was restored first, keeping the anterior part and the other side of the posterior as part of the original transitional overlay splint. The remaining two-thirds of the original transitional overlay splint was in place in the patient's mouth when making the impression. Using a face bow to mount on an articulator, the protrusive and lateral maxillomandibular relationship was recorded along with the inclination of the protrusive and lateral condylar guidance to accurately record the occlusal relationship. Thus, the central occlusal relationship, the anterior guidance, and the lateral movement were accurately recorded. In the second step, the anterior teeth were permanently restored using a face bow mounted on an articulator and a personalized anterior guidance disk to accurately copy the anterior guidance of the transitional overlay splint using the original protrusive and lateral condylar guidance. Thus, the maxillomandibular occlusal relationship and the anterior guidance can be accurately transformed from the transitional overlay splint to a fixed denture. This method requires only 6 subsequent visits, which is a convenience to patients. To obtain high masticatory efficiency and the restoration of facial appearance, to separate the space equally, and to recover the anterior guidance better because the maxillary and mandibular anterior teeth restoration is performed at the same time, the aesthetics are improved.

In the prosthodontic methods that restore the maxillary and mandibular jaws at the same time and to ensure that the occlusal spacing is divided equally, the following should be noted. If the bilateral posterior fixed restoration was made first, the splint that was adapted by the patients before cannot continue to be worn, and the anterior guidance built by the splint would be lost. If the anterior teeth are restored with a fixed restoration first, the occlusal relationship is not stable enough while wearing the remaining splint only in the posterior area. Even if the anterior guidance of the fixed restoration for the anterior teeth was an imitation of the transitional splint, it cannot be verified with any future completed posterior fixed reconstruction. By doing one side, posterior fixed restoration, first, guarantees the stability of the occlusal relationship between the restored posterior teeth and also retains the anterior guidance of the transitional splint. After the initial restoration of the first side a stable relationship can be established between the first side posterior reconstruction and the anterior guidance of the remaining two-thirds of the transitional splint. The anterior guidance of the second part of the anterior teeth fixed reconstruction provides checking and verification according to the transitional splint information. In the transitional trial period, adjust the occlusal contact points relationship between the maxillary and mandibular dentition as an anterior-posterior protected occlusion. The anterior teeth have no contact at the central occlusion, the bilateral posterior teeth have uniform contact, and the posterior teeth have no contact at the protrusive position. This occlusion contact was completely replicated during subsequent treatment (Figures 4(b), 7(b), and 8(a)).

## 4. Conclusion

For patients who need a whole fixed dentition reconstruction building an anterior guidance using a transitional splint, the first stage of the definite restoration involves the fixed reconstruction of one side of the posterior teeth and then a maxillary and mandibular anterior teeth restoration, and the final stage is on the other side of the maxillary and mandibular posterior restoration. The restoration according to this protocol can transfer the occlusal relation and the anterior guidance more precisely, reducing visit times, which is worthy of recommendation.
